# Recognizing Vocal Cord Dysfunction: Exercising Caution Before Intubation

**DOI:** 10.7759/cureus.46551

**Published:** 2023-10-05

**Authors:** Pamela Fernandes, Filip Siembida, Jaber Monla-Hassan, Daniel Bitetto

**Affiliations:** 1 Internal Medicine, Transitional Year Resident Program, Einstein Medical Center Montgomery, East Norriton, USA; 2 Medicine, Transitional Year Resident Program, Einstein Medical Center Montgomery, East Norriton, USA; 3 Pulmonology and Critical Care, Einstein Medical Center Montgomery, East Norriton, USA; 4 Hospital Medicine, Einstein Medical Center Montgomery, East Norriton, USA

**Keywords:** vcds, respiratory distress, unnecessary intubation, inducible laryngeal obstruction, vocal cord dysfunction

## Abstract

Vocal cord dysfunction (VCD) is the inappropriate adduction of the vocal cords during inhalation and sometimes, exhalation. Vocal cord dysfunction is often misdiagnosed in the emergency room as asthma exacerbation or laryngeal angioedema, leading to unnecessary and potentially harmful interventions including intubation and mechanical ventilation. Based on this, it is especially important to recognize this condition early to avoid intubation, which can further worsen VCD. This case presents a 74-year-old female with a history of hypertension and colon cancer who presented to the emergency department (ED) with respiratory distress associated with stridor and wheezing. Our literature review sheds light on identifying key clinical features, physical exam findings, diagnostic tests, existing treatment options for this condition, and preventive measures to minimize its occurrence.

## Introduction

Vocal cord dysfunction (VCD) syndrome, recently relabeled as inducible laryngeal obstruction (ILO), is a condition marked by inappropriate and transient vocal cord narrowing typically induced by external triggers. Both inspiration and expiration are affected, although the airway narrowing tends to be more pronounced during inspiration. Although this condition is more common in healthy, otherwise young females, it can also be associated with asthma and laryngopharyngeal reflux. Extubation from mechanical ventilation can trigger the syndrome [1]. Vocal cord dysfunction typically presents with symptoms such as wheezing, stridor, tachypnea, dysphonia, or cough and may be misdiagnosed as common airway diseases such as asthma flare-up, croup, laryngeal angioedema, or vocal cord palsy due to a similar presentation [2]. It is important to consider vocal cord dysfunction during the diagnostic process, as misdiagnosis can lead to intubation, which poses a risk of unnecessary mechanical ventilation complications. The purpose of this case report is to present a scenario in which vocal cord dysfunction was misdiagnosed, leading to mechanical ventilation, and to raise awareness of the syndrome as a potential differential diagnosis. Misdiagnosis leads to long-term morbidity.

## Case presentation

The patient is a 74-year-old female with a medical history of hypertension and metastatic colon cancer who presented to the ED with major respiratory distress. The patient recently underwent the removal of a spinal cord stimulator under moderate anesthesia at an outpatient surgical clinic. While in the recovery room, the patient developed a sore throat and began having acute shortness of breath, wheezing, and stridor. She received epinephrine and Benadryl before she arrived at the emergency department (ED).

Upon arrival at the ED, the patient was noted to be in major respiratory distress, with a respiratory rate in the 40s-60s and audible wheezing and stridor. She was placed on a non-rebreather mask and was maintaining adequate saturation. She was diagnosed with laryngeal angioedema and received additional epinephrine, steroids, and Benadryl without improvement in her symptoms. The patient ended up being intubated quickly after presentation by anesthesia using an endotracheal tube (ETT) instead of a bronchoscope, anticipating a difficult intubation. The patient had a similar presentation two years ago after a procedure that required intubation. She failed extubation multiple times with severe upper airway obstruction when evaluated by an ENT. When examined, she did not have any hypoxemia, and the anesthesiologist was called. They examined her vocal cords and did not find any anatomical abnormalities. Examination of the patient's upper airways during intubation did not show any edema of the vocal cords or supraglottic area. Her wheezing also immediately subsided after intubation.

**Figure 1:**
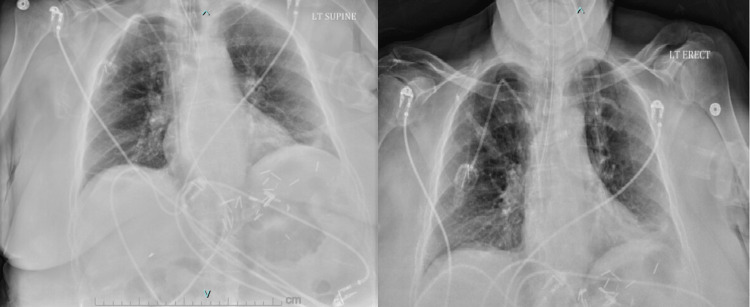
A normal chest X-ray shows no cardiopulmonary abnormalities.

The patient's peak airway pressure was 20cm of H_2_O and plateaued at 14cm of H_2_O, which ruled out any lower airway bronchospasm. The chest X-ray showed no significant abnormalities, as seen in Figure 1.

The patient had an elevated creatinine of 1.53 mmol/l with an unknown baseline creatinine. The white blood count (WBC) was measured at 7.1 without eosinophilia. The patient was transferred to the intensive care unit (ICU). Upon arrival, her vitals were stable with the patient sedated on propofol. A physical examination did not reveal any abnormalities.

The patient was suspected to have vocal cord dysfunction given the lack of laryngeal edema and absence of airway resistance abnormalities post-intubation. After talking to the patient’s family, the ICU team was able to extract a history of a similar episode two years before this presentation, which required prolonged mechanical ventilation and was associated with repeated re-intubation, from probable VCD. Based on the high probability of VCD, the ICU team decided to extubate the patient after placing the patient on a de-escalating low-dose dexmedetomidine infusion (0.6 mcg/kg/hour) and receiving a low dose (0.5 mg) of IV Ativan. The patient did not receive any additional therapy for asthma or laryngeal angioedema while in the ICU thereafter. The patient did well during a short spontaneous breathing trial and was successfully extubated with coaching after three hours of being on mechanical ventilation. After extubating, there was no residual stridor, wheezing, or dyspnea.

The patient was monitored in the ICU overnight after extubation. She remained stable the next morning with normal oxygen saturation in room air and a normal physical exam. The patient was noted to have a chronic hoarse voice. She was ready to be discharged home with probable vocal cord dysfunction syndrome. She was educated about her condition and advised to follow up with a speech therapist for breathing techniques in case she developed another episode in the future.

## Discussion

Vocal cord dysfunction is an obstruction of the upper airway due to inappropriate transient paradoxical adduction of the vocal cords, typically in response to external triggers affecting mostly inspiration but sometimes both inspiration and expiration. The exact prevalence of vocal cord dysfunction (VCD) is not well defined due to several factors, including the challenges in diagnosing the condition accurately. Vocal cord dysfunction can often mimic other respiratory conditions like asthma, laryngeal angioedema, and vocal cord paresis; therefore, misdiagnosis or underdiagnosis is quite common. It can occur at any age, but it is more common in adolescents and young adults. It has been reported to be more prevalent in females than males [3]. Vocal cord dysfunction can sometimes occur in athletes or those who engage in regular physical activity. The exercise-induced triggers may exacerbate VCD symptoms. Individuals who require extensive voice use, such as teachers, singers, and healthcare professionals, might be more susceptible to developing voice-related issues, including VCD. However, this does not necessarily mean that VCD is exclusive to these professions. Vocal cord dysfunction symptoms can be triggered by stress, anxiety, strong odors, irritants, or physical touching of the upper airways with an endotracheal tube (ETT) or laryngeal mask airways (LMA) during surgical procedures or post-extubation. Vocal cord dysfunction can sometimes coexist with other conditions, such as asthma, gastroesophageal reflux disease (GERD), and anxiety disorders.

Pathophysiology

The pathophysiology of VCD involves the abnormal movement of the vocal cords during the breathing cycle. Normally, the vocal cords abduct and widen the laryngeal opening during inspiration to maximize the airflow into the lungs. The vocal cords adduct mildly and slightly narrow the airway during exhalation to prevent air from escaping too quickly. During a VCD episode, there is an inappropriate and transient vigorous adduction of the vocal cords constricting the laryngeal opening, which is more pronounced during inspiration, resulting in breathing difficulties and other related symptoms.

The exact underlying causes of VCD are not fully understood, but several factors can contribute to its development. It often involves a combination of physiological, psychological, and environmental factors. One of these factors is muscle dysfunction. The intrinsic laryngeal muscles can become hyperactive or spasmodic, leading to improper closure during inhalation. This can be triggered by numerous factors, including stress, anxiety, and abnormal neuromuscular responses. Neurological factors can also contribute to VCD. Dysfunctional nerve signals controlling the laryngeal muscles can contribute to VCD. An imbalance in the coordination between the muscles involved in breathing and vocal cord movement may occur. Psychological factors such as stress, anxiety, and emotional factors can trigger or exacerbate VCD symptoms [4]. Stress-induced muscle tension and altered breathing patterns can also cause VCD. Exercise-induced factors such as physical activity, particularly intense or strenuous exercise, can trigger VCD. This may involve the overuse of accessory muscles for breathing, leading to vocal cord closure during inhalation. Upper airway irritation due to exposure to irritants, allergens, strong odors, and respiratory infections can irritate the upper airway and contribute to vocal cord dysfunction. Gastroesophageal reflux disease and laryngopharyngeal reflux (LPR) can lead to irritation of the laryngeal area, potentially affecting vocal cord function. Individuals with VCD may have heightened sensitivity in their upper airways, making them more prone to various triggers [5]. Individuals with VCD may also develop abnormal breathing patterns, such as shallow or rapid breathing, which can contribute to vocal cord dysfunction.

The interplay of these factors can lead to the characteristic symptoms of VCD, including inspiratory stridor, wheezing, throat discomfort, and voice changes. It is important to note that the pathophysiology of VCD is complex and can vary among individuals.

Clinical presentation

Some common signs and symptoms of vocal cord dysfunction include breathing difficulties such as stridor due to the restricted movement of the vocal cords and inspiratory wheezing during inhalation, although wheezing can be heard in both inspiration and expiration, which can mimic asthma. Another common symptom is voice changes, which include hoarseness characterized by a rough or raspy voice, vocal fatigue due to the extra effort required to overcome restricted vocal cord movement, a feeling of choking or throat tightness, the sensation of a "lump" in the throat or a feeling of choking even when not eating or drinking, and a chronic or persistent cough that may not respond to traditional cough treatments. Symptoms may occur episodically, often triggered by factors such as exercise, stress, strong odors, allergens, frigid air, exposure to irritants, or physically touching the vocal cords with an endotracheal tube or laryngeal or laryngeal mask airways during surgical procedures or post-extubation, as happened in our case. Vocal cord dysfunction symptoms can lead to feelings of anxiety, panic, or distress due to difficulty breathing. Breathing patterns may become rapid and shallow due to the sensation of restricted airflow. Symptoms tend to worsen during physical activity, leading to exercise intolerance. Some individuals with VCD may experience relief from symptoms when they hold their breath. In some cases, symptoms may occur at night. VCD symptoms can overlap with other respiratory conditions, such as asthma or LPR.

Diagnosis

Knowledge of the existence of VCD is key. A high index of clinical suspicion based on the history, symptoms, and signs is the first step toward establishing the diagnosis of VCD. If the chest X-ray is normal, as was in our case (Figure 1), then the next step would be to use advanced imaging. One of the essential tools used in VCD is flexible laryngoscopy, which helps to visualize the movement and function of the vocal cords during breathing and speaking and confirm the abnormal movements during VCD episodes [6]. It is the diagnostic gold standard test to diagnose paradoxical adduction of the vocal cords. It can also identify the presence or absence of other vocal cord conditions, such as laryngeal edema, vocal cord paresis, or vocal cord lesions or lumps often seen in upper airway obstructive disease. Stroboscopy, a specialized form of laryngoscopy, uses strobe light to create slow-motion images of vocal cord vibration, which can help identify irregularities in movement. Flow-volume loops of bedside spirometry can also depict airflow abnormalities, typically flattening of the inspiratory or both inspiratory and expiratory limbs suggestive of VCD. The methacholine challenge test can assess for hyperresponsiveness of the airways, often seen in conditions like asthma. If vocal cord dysfunction is present, symptoms may worsen during the test and lead to the classic flow-volume loop upper airway obstructive pattern. Exercise challenge tests, such as running on a treadmill or doing specific breathing exercises while being monitored for changes in vocal cord function, can be done. The provocative inhalational challenge, where an irritant or allergen is inhaled to induce symptoms, can help confirm the diagnosis when symptoms are reproduced. Esophageal pH monitoring can rule out GERD as a potential cause of symptoms. Gastroesophageal reflux disease can sometimes mimic VCD. Acoustic and perceptual voice analysis can help assess voice quality and any changes related to vocal cord dysfunction.

Management

Therapeutic breathing maneuvers and techniques to relax the vocal cord are considered first-line therapy for vocal cord dysfunction-associated dyspnea [3]. They include progressive muscle relaxation and deep breathing, or diaphragmatic breathing. The latter requires participants to contract the diaphragm, slowly inhaling and exhaling. Deep breathing increases blood oxygen levels and stimulates the vagus nerve. When used with guided imagery and sensory engagement, it can reduce the stress and anxiety often seen in VCD. Breathing retraining exercises taught by a respiratory therapist or speech therapist can help individuals learn proper breathing patterns to reduce the likelihood of vocal cord dysfunction episodes triggered by improper breath control.

Speech therapy is a common approach for managing vocal cord dysfunction and helping prevent future attacks. A speech-language pathologist can work with individuals to teach them proper breathing techniques, relaxation exercises, and strategies to control and coordinate vocal cord movement. Optimizing the treatment of known coexistent conditions such as asthma or allergies helps alleviate VCD by reducing the triggers. In severe cases such as the ones associated with dysphonia, injections of botulinum toxin (Botox) into the vocal cords can temporarily weaken overactive vocal cord muscles, thus alleviating symptoms [7]. Since stress and anxiety can exacerbate vocal cord dysfunction, counseling or psychotherapy sessions with a psychologist or counselor may help individuals manage emotional triggers and prevent VCD episodes. Anxiolytics, when indicated, can help control VCD. In exceedingly rare and severe cases of vocal cord dysfunction that does not respond to other treatments, surgical interventions such as thyroarytenoid myoneurectomy or laryngeal nerve reinnervation might be considered [8]. These are typically last-resort options.

In the ED, it is important to be vigilant and consider VCD as a differential diagnosis when there is stridor, upper airway wheezing, or voice changes in the appropriate clinical scenario. Careful flexible laryngoscopy to inspect the movement of the vocal cords and to rule out other vocal cord abnormalities such as laryngeal edema or vocal cord paresis is a crucial tool to abort intubation in these individuals, which can lead to unnecessary prolonged mechanical ventilation and its complications [9]. A comparison of the most common differential diagnoses of VCD is listed in Table 1 below.

**Table 1:** Comparison of asthma, LPR, and VCD LPR: laryngopharyngeal reflux; VCD: vocal cord dysfunction

Characteristic	Asthma	Laryngopharyngeal reflux (LPR)	Vocal cord dysfunction (VCD)
Primary symptoms	Wheezing, coughing, chest tightness, and shortness of breath	Chronic throat clearing, hoarseness, and globus sensation (feeling of a lump in the throat)	Inspiratory stridor, inspiratory wheezing, choking sensation, voice changes
Triggers	Allergens, exercise, cold air, respiratory infections, and irritants	Spicy foods, acidic foods and drinks, and lying down after eating	Exercise, stress, strong odors, and irritants
Symptoms during exercise	Can worsen during or after physical activity	May or may not worsen during exercise	Typically worsens during exercise
Voice changes	Not a primary symptom	Hoarseness possible	Hoarseness, vocal fatigue
Cough characteristics	Dry or productive cough, especially at night	Chronic cough, often dry	Coughing may be present
Response to bronchodilators	Often effective in relieving symptoms	Not effective in treating symptoms	May not be effective
Gastrointestinal symptoms	Not a primary feature	Possible heartburn or regurgitation	Not typically associated
Breathing difficulty	Expiratory wheezing may be present	Not a primary symptom	Inspiratory stridor, choking sensation
Diagnostic tests	Spirometry, peak flow measurements, and bronchial challenge tests	Laryngoscopy, pH monitoring	Laryngoscopy, pulmonary function tests, and exercise challenge
Treatment approach	Bronchodilators, corticosteroids, and avoiding triggers	Lifestyle changes, dietary modifications, and antacids	Speech therapy, breathing exercises, and relaxation techniques
Specialists involved	Pulmonologist, allergist	Gastroenterologist, otolaryngologist	Otolaryngologist, speech therapist

Our patient had a history of post-procedure prolonged mechanical ventilation caused by VCD misdiagnosis. Administering a mixture of helium and oxygen (Heliox) reduces airway resistance and may result in the de-escalation of VCD symptoms [10]. Judicial use of anxiolytics in the ED during VCD flare-ups with major respiratory distress can help reverse the condition [11].

## Conclusions

In conclusion, healthcare professionals must be vigilant in recognizing VCD, as it is often misdiagnosed in the clinical setting. The condition is often mistaken for asthma or worsening respiratory status, and by identifying key clinical features, performing thorough physical exams, and performing certain diagnostic tests, VCD can be diagnosed appropriately. A timely diagnosis can lead to the necessary treatment and prevention of adverse outcomes associated with mechanical ventilation. Once identified, pursuing speech therapy is essential, as it can assist a patient with managing a future attack. Moreover, implementing preventive strategies such as sessions with a psychiatrist to prevent potential triggers is a crucial part of the management of VCD.
